# Integrity

**Published:** 1862-10

**Authors:** W. H. Atkinson


DENTAL REGISTER OF THE WEST.

VOL. XVI.] OCTOBER, 1862. [No. 10.

### Original Essays and Communications.

### INTEGRITY.

Read before the Brooklyn Dental Association.

BY DR. W. H. ATKINSON.

TOGETHERNESS facilitates accuracy of measurement, and concentrates ability to the one or many purposes of life; while apartness diffuses, conceals, and renders uncertain and unsatisfactory all attempt at definite conclusion or precision of principle, dissipates ability, and militates against efficiency.

A purposeless life is a living death to any sentient being. Lowness of purpose marks low on the scroll of use or fame, while elevated purpose inaugurates greatness of character, integral and reputed.

He who has habitually indulged in high and ennobling views of his consequence and mission on earth has so far outgrown common-place habits of thought, word and deed, that all these are tame and trite to him, not seeming worth the performance, for the meager prospect of good they promise. And hence the bond between him and the masses can only be cemented when they are wrought up to the appreciation of his standard by great occasions, or through illimitable goodness of heart in him toward them, holding them by sympathy within the charmed circle, till what had seemed cant and self-love is proved to be love for them.

Genuine integrity of character will soon distinguish any one bold enough to live it out in his daily life.

There is the integrity of fossiliferous consistency, and thank God, there is, too, the integrity of love of truth, at whatever cost of money, reputation, limb or life itself, in the little and fearful selfish sense.

He who has the integrity of obedience to his highest conception of good at all times, under all circumstances, is really the only truly integral man! no matter how misunderstood, misrepresented, despised and spat upon he may be by the poor pigmies who dupe themselves or allow others to dupe them into the weakness of thinking they are really doing God service, and carelessly or purposely neglecting to apply the rule that "he can not be wrong whose life is in the right."

The really integral man has no time to waste in puerile explanation, while the continued pursuit of unbroken life-purpose urges him to accomplish the greatest good in the shortest time possible, which in its turn fortunately proves the very best possible explanation of all seeming error or half statement of truth. These being but the gathering up of our energies to express ourselves so that the great intendment of life may be made to appear. All these misapprehensions of our meaning hold a negative place in the activities of life, and correspond to the diastole of the heart, without which the systole could never send the red life currents on their mission of irrigation and renovation of all the primates of the integral system.

Integrity—wholeness—from the prim. "in," and "tango" to touch—hence untouched or unscathed, unfractionalized, all sided, not one sided—so strict integrity, morally, mentally and physically, is very desirable, and especially requisite to the production of the highest type of professional excellence in individual or associated capacity!

To be of true integrity, it behooves us to be integrally built from the commencement. An integral dentist, then, will be he who embodies all that pertains to the requisite knowledge and skill to fit him for complete diagnosis and execution. This being so, he who lays claim to integrity as a dentist has assumed heavy responsibility. I do not say we should shrink from it because it demands so much of us, but in view of the immense mischief we shall perpetrate without it, we are bound to abandon the practice, or strive for the fullest integrity conceivable or attainable; for whatever is once conceived in the true spirit of philosophic love is already half accomplished, as evinced particularly in the manifold necessities of the dentist, scarcely having time to find expression before the need is supplied as by anticipative inspiration in one or many, which has so often been the cause of charges of plagiarisms and a stealing of each others' thunders, as is frequently said by those devoid of integrity on the one hand, or broad intelligence of the legitimate modes of progression on the other.

Be well assured we have more genuine moral integrity as a body than we ordinarily accord to each other. Many, doubtless, are entrapped into claiming or seeming to claim for themselves the discoveries and inventions of others, from the fact that almost every one has felt his necessities insidiously leading him into universal views, so akin to his fellows as to require closer scrutiny than most of us exercise to avoid the blunder of confounding them as identical.

What we need most is frequent, free and fraternal contacts of spirit, soul, and bodily presence, to level us all up to the standard of the highest integralism possessed by the broadest, the brightest and best among our advanced and advancing body.

